# The Impact of Pneumococcal Conjugate Vaccine (PCV) Coverage Heterogeneities on the Changing Epidemiology of Invasive Pneumococcal Disease in Switzerland, 2005–2019

**DOI:** 10.3390/microorganisms9051078

**Published:** 2021-05-18

**Authors:** Oluwaseun Rume-Abiola Oyewole, Phung Lang, Werner C. Albrich, Kerstin Wissel, Stephen L. Leib, Carlo Casanova, Markus Hilty

**Affiliations:** 1 Institute for Infectious Diseases, University of Bern, 3001 Bern, Switzerland; oluwaseun.oyewole@ifik.unibe.ch (O.R.-A.O.); stephen.leib@ifik.unibe.ch (S.L.L.); carlo.casanova@ifik.unibe.ch (C.C.); 2 Graduate School for Cellular and Biomedical Sciences, University of Bern, 3001 Bern, Switzerland; 3 Epidemiology, Biostatistics and Prevention Institute, University of Zürich, 8001 Zürich, Switzerland; phung.lang@uzh.ch; 4 Division of Infectious Diseases and Hospital Epidemiology, Cantonal Hospital St. Gallen, 9007 St. Gallen, Switzerland; werner.albrich@kssg.ch (W.C.A.); kerstin.wissel@checkpoint-zh.ch (K.W.); 5 Swiss National Reference Center for Invasive Pneumococci (NZPn), Institute for Infectious Diseases, University of Bern, 3001 Bern, Switzerland

**Keywords:** *Streptococcus pneumoniae*, capsular polysaccharide, pneumococcal conjugate vaccines (PCVs), Switzerland, serotype 3

## Abstract

Pneumococcal conjugate vaccines (PCVs) have lowered the incidence of invasive pneumococcal disease (IPD) worldwide. However, the influence of regional vaccine uptake differences on the changing epidemiology of IPD remains unclear. We aimed to examine the overall impact of both seven- and 13-valent PCVs (PCV7 and PCV13) on IPD in Switzerland. Three-year periods from 2005–2010 and 2011–2019 were considered, respectively, as (early and late) PCV7 eras and (early, mid and late) PCV13 eras. Vaccine coverage was estimated from a nationwide survey according to east (German-speaking) and west (French/Italian-speaking) regions for each period. Reported incidence rate ratios (IRRs) were compared between successive periods and regions using nationwide IPD surveillance data. Overall IPD incidence across all ages was only 16% lower in the late PCV13 era compared to the early PCV7 era (IRR 0.83, 95% CI 0.79–0.88), due to increasing incidence of non-PCV-type IPD (2.59, 2.37–2.83) in all age groups, except children <5 years. PCV uptake rates in swiss children were slightly higher in the west than the east (*p* < 0.001), and were accompanied by lower IPD incidences across all age groups in the former region. Post-PCV13, non-PCV serotypes 8, 22F and 9N were the major cause of IPD in adults ≥65 years. Increased PCV coverage in both areas of Switzerland resulted in a decrease in vaccine-type and overall IPD incidence across all age groups, in a regionally dependent manner. However, the rising incidence of non-vaccine-type IPD, exclusive to older adults, may undermine indirect beneficial effects.

## 1. Introduction

Invasive pneumococcal disease (IPD), comprising of pneumococcal pneumonia, bacteremia and meningitis, is a leading cause of mortality in children <5 years, adults ≥65 years and immunocompromised individuals. In 2005, the world health organization (WHO) estimated 1.6 million annual IPD-related deaths, of which 0.7–1 million were in children <5 years [1]. Since the introduction and widespread implementation of pneumococcal conjugate vaccines (PCVs), especially in children <5 years, large reductions in the burden and incidence of IPD have been observed in vaccinated children worldwide, as well as an indirect herd protection effect among adults and unvaccinated individuals [2,3].

In Switzerland, the heptavalent pneumococcal conjugate vaccine (PCV7, Prevenar^®^, Pfizer), which covered serotypes 4, 6B, 9V, 14, 18C, 19F and 23F, was first recommended in 2001 for children <5 years with increased health risk for IPD, following a 3 + 1 dosing schedule at 2, 4 and 6 months of age and a booster at 12–15 months [4]. In late 2005, PCV7 was additionally recommended as a supplementary (optional) vaccination for healthy children <2 years, following a 2 + 1 dosing schedule at 2, 4 and 12 months with no catch-up campaign [4]. Since late 2010, the thirteen-valent pneumococcal conjugate vaccine (PCV13, Prevenar13^®^, Pfizer), which covers the PCV7 serotypes as well as six additional serotypes (1, 3, 5, 6A, 7F, and 19A), has replaced PCV7 with a catch-up campaign extended up to 5 years of age [5]. In 2014, PCV13 became the only recommended pneumococcal vaccine, replacing the 23-valent pneumococcal polysaccharide vaccine (PPV23, Pneumovax^®^23, Merck) [5], which had been implemented since 2000 for individuals ≥5 years at higher risk for IPD and adults ≥65 years, but had only shown a moderate protection efficacy compared to PCV13 [6,7].

The impact of targeted pneumococcal vaccination programs on IPD has been investigated in different countries [8,9,10]. However, little is known about the influence of regional vaccine uptake differences on IPD epidemiology. We have previously shown higher (up to five times) pneumococcal serotype-specific antibiotic resistance rates in western Switzerland (French-speaking) than the east (mostly German speaking) and attributed these to a higher (up to 1.5 times) antibiotic consumption in the former [11,12]. Variations have also been reported for methicillin-resistant staphylococcus aureus (MRSA) epidemiology and human papillomavirus (HPV) vaccine uptake among the different language speaking regions in Switzerland [13,14]. Switzerland therefore provides a unique setting for studying regional variations in pneumococcal vaccine uptake and its association with serotype epidemiology.

In this study, we investigate the impact of PCV introduction on IPD in Switzerland and its regions during both PCV7 and PCV13 eras from 2005 to 2019 using nationwide IPD and vaccination survey data. Our two objectives are (i) an overall analysis of IPD incidence and serotype distribution in Switzerland during the PCV eras, and (ii) an assessment of regional (east/west) variations in PCV coverage and its association with both age-specific and overall IPD incidence. 

## 2. Materials and Methods

### 2.1. Data Collection

In Switzerland, the Federal Office of Public Health (FOPH) has mandated all clinical microbiology laboratories to send invasive *Streptococcus pneumoniae* isolates from sterile body sites (blood; cerebrospinal fluid (CSF); pleural, joint and peritoneal fluid) to the National Center for Invasive Pneumococci (NZPn). Isolates were received with corresponding information on the patient’s age, gender and canton of origin. It is estimated that approximately 90% of all invasive isolates received at NZPn can be linked to physician-reported IPD cases [15]. At the NZPn, isolates were cultured on Columbia sheep blood agar plates and *S. pneumoniae* was confirmed using conventional methods (alpha hemolysis, optochin susceptibility and bile solubility). The confirmed *S. pneumoniae* isolates were then assigned serotypes based on the Quellung reaction.

The Swiss National Vaccination Coverage Survey (SNVCS) is a cross-sectional survey which has been monitoring immunization coverage of children and adolescents (2-, 8- and 16- year-olds) across the 26 Swiss cantons every three years since 1999 [16]. Methodology of data collection has been previously described [16]. In brief, information on children and their parents were requested from the municipalities or central office of registry. Children from each age group were randomly selected via simple random or cluster sampling. Families of selected children were contacted up to three times by mail and once by telephone, if permitted by the cantonal authorities, and asked to mail a copy of the children’s vaccination card or upload it onto an online platform developed for the study. Primary care physicians were contacted when vaccination data were unclear. Three cantons transmitted vaccination data for 8- and 16-year-olds collected by their school nurses. One canton did not collect vaccination data for 8-year-olds. Vaccination information that was not received through either of the aforementioned methods was excluded from the survey database. Pneumococcal vaccination data for 2-year-olds during 2008–10, 2011–13, 2014–16 and 2017–19, were included in the study with cumulative survey response rates of 80.5%, 77.4%, 72% and 68.2%, respectively. For 8-year-olds, only pneumococcal vaccination data during the last two survey periods, 2014–16 and 2017–19, were included with response rates of 70.9% and 68.2%, respectively. Data from earlier survey periods were excluded as PCV use was still not widespread for the respective age groups during those periods. Data was anonymized prior to statistical processing.

### 2.2. Case Definition and Regional Assignment

An IPD case was defined as a patient from whom an invasive isolate was confirmed by the NZPn. Information on the isolates used in this study were not linked to physician cases. Hence, these counts do not represent the absolute numbers of IPD cases monitored by the FOPH in Switzerland. 

Western Switzerland was considered as primarily French and Italian speaking cantons, which includes cantons Fribourg, Geneva, Jura, Neuchatel, Vaud, Valais and Ticino (Figure S1). The remaining 19 primarily German speaking cantons were considered eastern Switzerland. Isolates with missing residence canton information (~16%) were assigned to the canton region of the referring (local) laboratory, where possible. 

### 2.3. Statistical Analyses

Annual IPD incidences during 2005–2019 were calculated overall and for age groups (< 5, 5–64 and ≥65 years), using the yearly number of IPD isolates as the numerator and corresponding annual population estimates, obtained from the Swiss Federal Statistics Office (https://www.bfs.admin.ch/bfs/de/home/statistiken/bevoelkerung.html, accessed on 10 December 2020), as the denominator. Regional incidences were calculated based on previously defined cantonal groupings. For overall analysis, incidence rate ratios (IRRs) were estimated to allow pairwise comparisons between the early PCV7 (2005–2007), late PCV7 (2008–2010), early PCV13 (2011–2013), mid PCV13 (2014–2016) and late PCV13 eras (2017–2019). For regional analysis, IRRs between regions were compared using the east as reference. Incidence and rate ratios were calculated using the poisson exact test (R package epitools v0.5–10.1) and rate ratio function (R package fmsb v0.7.0) respectively.

Annual serotype distribution proportions were estimated for individual serotypes and stratified by PCV-type (PCV7, PCV13nonPCV7 and non-PCV) and age groups. Trend analyses were performed using the Chi-squared test for trend in proportions. Annual serotype incidences were calculated overall and for regions by multiplying annual serotype (individual or PCV-type) proportions by the annual incidence.

As previously described, vaccination survey data were first weighted to account for sampling design and adjusted for non-response [16]. Vaccination coverage estimates and 95% confidence intervals (95% CI) for the Swiss population were then calculated for each region per PCV era using the survey option in Stata (v13.1). Timeliness of vaccine uptake was plotted using the cumulative proportion of administered doses by age. Regional differences in vaccine coverage proportions were estimated with the two-proportion z-test. For all analyses, *p* < 0.05 was considered significant. Excluding vaccination coverage estimates, all other analyses were performed using R (v3.6.1) in the RStudio environment (v1.2.1335). 

## 3. Results

### 3.1. Characteristics of IPD from 2005–2019

In total, 14680 laboratory-confirmed isolates of invasive pneumococcal disease were reported in Switzerland during 2005–2019. Of these, the majority (92.6%) of *S. pneumoniae* were isolated from blood, 2.1% from CSF and the remaining from other diverse body sites. The absolute number of invasive isolates increased from 1016 in 2005 to 1129 in 2008 and steadily decreased to 877 in 2016 (Table S1). Total invasive isolates then spiked up to 1012 in 2017 before falling to 915 in 2019. In total, 4.9% (*n* = 714) were collected from children aged <5 years, 36.5% (*n* = 5355) from those aged 5–64 years and 52.5% (*n* = 7707) from adults ≥65 years (Table S1). Overall, 904 (6.2%) isolates had missing data for age.

### 3.2. Overall Impact of PCV on IPD Incidence and Serotype Distribution in Switzerland

**Figure 1:**
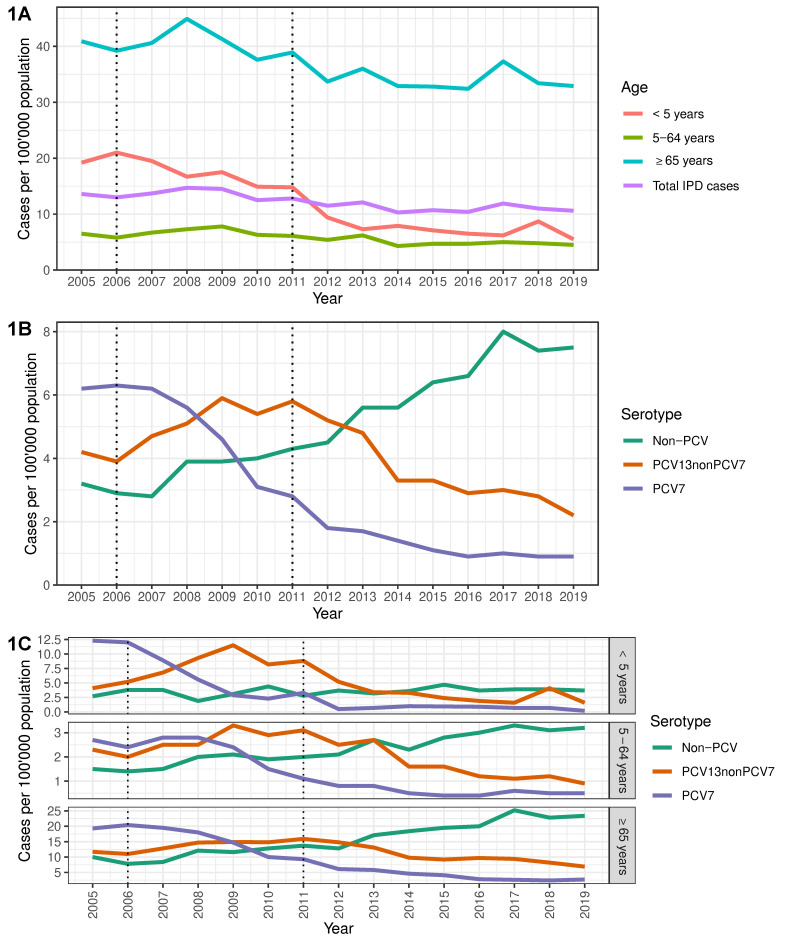
Incidence of invasive pneumococcal disease in Switzerland during 2005–2019. Overall incidence stratified by (**A**) age group, (**B**) vaccine serotype and (**C**) age group and vaccine serotype. Introduction of PCV7 (2006) and PCV13 (2011) are indicated by dotted black lines.

After the introduction of PCV7 in 2006, overall IPD incidence slightly decreased from a peak of 14.1 cases in 2008 to 12.5 cases per 100.000 population in 2010. Following PCV13′s implementation in 2011, IPD incidence further decreased to 10.4 cases per 100.000 population in 2016, followed by a temporary rebound in 2017, which was more pronounced among adults aged ≥65 years (Figure 1A). In subsequent years, the rebound in total cases declined (Figure 1A). 

When comparing PCV eras, strong effects of PCV7 were observed during the late PCV7 era (2008–2010), evidenced by the lower PCV7-type IPD incidence across all age groups (Figure 1B; Table 1). This was more pronounced in children <5 years, where the decline was 68% lower compared to the early PCV7 era (2005–2007) (Figure 1C; IRR 0.32, 95% CI 0.23–0.46). 

After PCV13 introduction, incidence of PCV7-type IPD continued to decline and by the late PCV13 era had decreased 85% across all ages compared to the early PCV7 era (Table 1). However, overall IPD incidence was only 16% lower, as non-PCV-type IPD cases continued to increase among age groups ≥ 5 years (Table 1; Figure 1C). No significant decreases in total IPD incidence were observed for age groups, when comparing mid- and late-PCV13 eras (Table 1).

**Figure 2:**
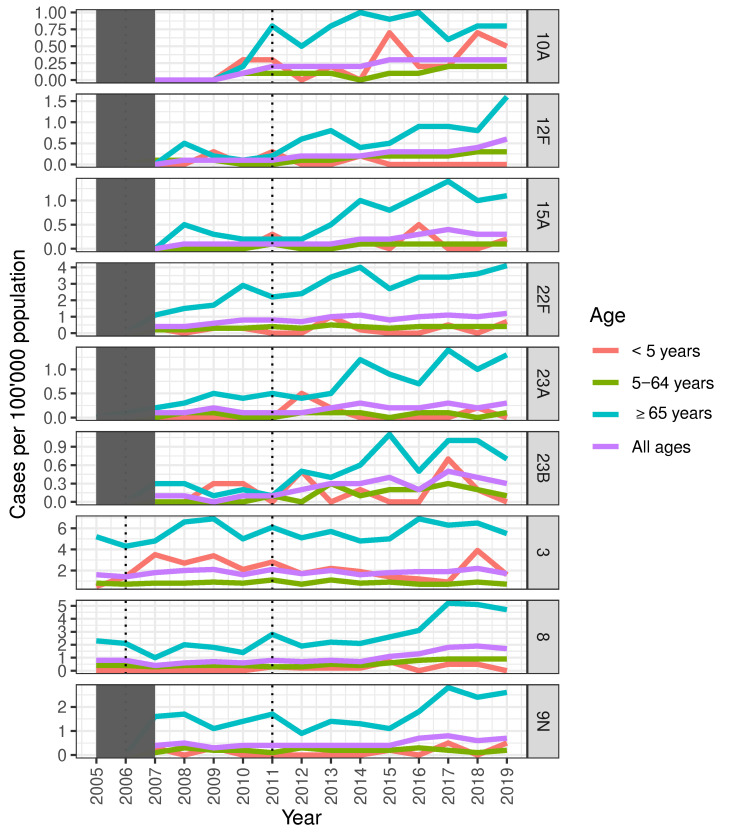
Single Serotype analyses in Switzerland, 2005–2019. Single serotypes with high IPD incidences are indicated. Certain serotypes have not been part of the routine typing in Switzerland for 2005–2006 (hence the data is shaded). Introduction of PCV7 (2006) and PCV13 (2011) are indicated by dotted black lines.

Of individual vaccine serotypes (VTs), incidence of serotype 3 IPD did not decrease after PCV13 introduction and remained predominantly high among adults ≥65 years (Figure 2; Table S2). In addition, non-vaccine serotypes (NVTs) which increased post-PCV13 introduction were most prevalent in adults ≥65 years (Table 1; Figure 2). Of these, serotypes 8, 22F and 9N contributed the highest IPD incidence (Figure 2). By the end of 2019, NVTs were responsible for 70% of all IPD cases compared to 21% by PCV13nonPCV7 and 9% by PCV7 serotypes (Table S2).

### 3.3. Overall Impact of PCV on IPD Incidence in Eastern and Western Switzerland

**Figure 3:**
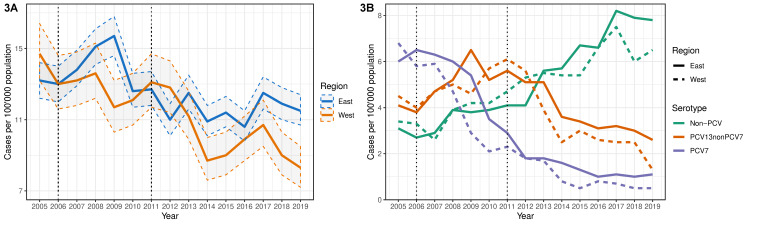
Incidence of invasive pneumococcal disease by region in Switzerland, 2005–2019. Regional incidence is illustrated (**A**) overall and (**B**) stratified by vaccine serotype. 95% confidence intervals are depicted as the shaded region between ribbon dashed lines. Introduction of PCV7 (2006) and PCV13 (2011) are indicated by dotted black lines.

The majority of IPD isolates were reported in the eastern (*n* = 10600; 72.2%) as compared to the western region (*n* = 4067; 27.7%) and unknown origin (*n* = 13; 0.09%). Within the two Swiss regions, IPD incidence again showed a declining trend during 2005–2019, which was mostly lower in the west compared to the east, except for the early PCV7 and early PCV13 eras (Table 2; Figure 3A). After the early PCV13 era, incidence of VT-type IPD continued to decrease in both regions, though more significantly in the west than the east, whereas non-PCV-type IPD showed a higher significant increase in the east than the west during the late PCV13 era (Table 2; Figure 3B).

### 3.4. Pneumococcal Vaccine Coverage in National and Regional Switzerland

**Figure 4:**
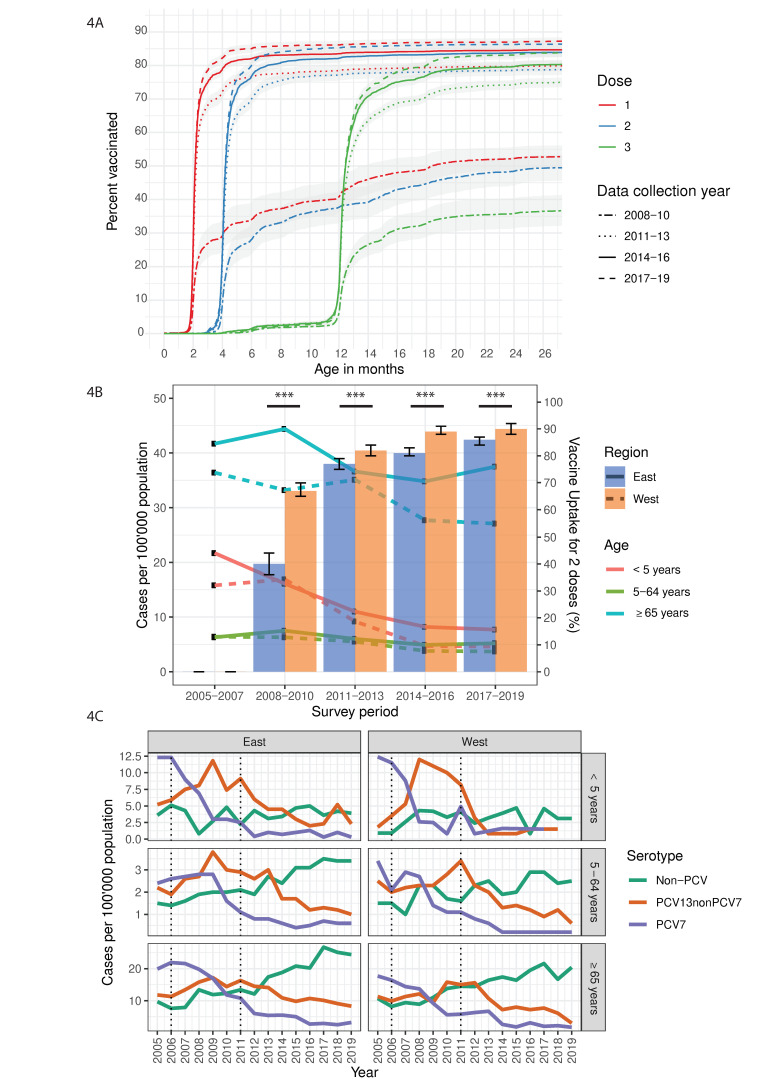
Timeliness of pneumococcal conjugate vaccine uptake, regional estimates of coverage and age-group specific incidence in Switzerland, 2005–2019.(**A**) Timeliness of vaccine uptake in children aged 2-years, vaccinated with 1–3 doses of PCV7 or PCV13, (**B**) age group-specific IPD incidence and regional estimates of pneumococcal vaccination coverage among children aged 2-years with 1–2 PCV doses, and (**C**) age group- and region-stratified IPD incidence by vaccine serotypes. 95% confidence intervals are depicted by (A) shaded regions and (B) error bars. For the combined graph (B), the primary Y-axis shows the IPD cases per 100′000 population (line graph) and the secondary Y-axis shows the vaccine uptake rates (bar graph). Introduction of PCV7 (2006) and PCV13 (2011) are indicated by dotted black lines (C). Proportions and vaccination coverage estimate during the 2005–2007 period were excluded due to very low rates (<3%). *** indicates *p* < 0.001 (Two proportions z-test).

Among children aged 2 years-old (target population), who were vaccinated with at least one PCV dose, uptake rates increased from 53% (95% CI, 49.6–56.4%) during the late PCV7 era to 88% (86.8–88.9%) during the late PCV13 era (Figure 4A). Similar upward trends in the timeliness of PCV uptake were noted for second and third doses (Figure 4A). When stratified by region, pneumococcal vaccine coverage rates increased in the east from 43%, 40% and 26% during the late PCV7 era to 87%, 86% and 83% during the late PCV13 era for doses one, two and three in the target population, respectively (Figure 4B; Figure S2A,B). In the west, coverage rates were significantly higher (*p* < 0.001) and increased from 71%, 67% and 57% during the late PCV7 era to 91%, 90% and 80% during the late PCV13 era for doses one, two and three in the target population, respectively (Figure 4B; Figure S2A,B). Among 8-year-old children selected for the vaccine uptake surveys, similar regional differences were also observed during the last two PCV eras (Figure S3A–D).

### 3.5. Indirect and Overall Impact of PCV Coverage on IPD Incidence in Age Group Populations in the East and West

We next stratified our IPD incidence data according to the east/west region and respective age groups. We hypothesized that increased PCV coverage may be associated with decreased IPD incidence, either through a ‘direct effect’ (in the children <5 years) or an ‘indirect effect’ (mainly for adults ≥65 years).

For the early PCV7 era (2005–2007), our hypothesis was unsupported, as we observed lower IPD incidences among age group extremes (children <5 years and adults ≥65 years), despite extremely low vaccine coverage rates of this era (Figure 4B; Supplementary Tables S3–S5). However, given that such regional differences were not observed for overall IPD incidence, caution should be taken when interpreting this age group-stratified result, especially since a higher proportion of unknown age group cases were observed in the west compared to the east during this period.

In the late PCV7 era (2008–2010), support for our hypothesis was corroborated by higher PCV7 uptake rates in the west and a significant decrease in the IPD incidence of adults ≥65 years in this region, possibly as an indirect effect of the higher vaccine coverage in the west (Figure 4B; Table S5). In the ensuing (PCV13) era (2011–2013), IPD incidence remained lower in the west, albeit not significantly, as incidence proportions sharply decreased in the east, especially among adults ≥65 years, probably due to a 92.5% increase in PCV coverage (2 doses) in the east, which minimized coverage differences between both regions and the incidence gap between aged adults in these regions (Figure 4B).

However, during the mid- and late- PCV13 eras, increasing incidence of non-PCV-type IPD was observed in both regions, where they rose 78% among adults ≥65 years living in the east, compared to 30% among those in the west, widening the incidence gap between both regions (Figure 4B,C; Table S5).

## 4. Discussion

In this population-based study, we examined the overall impact of PCV7 and PCV13 introduction on IPD in Switzerland as well as the influence of PCV coverage heterogeneities in the country’s largely German (east)- and French/Italian (west)- speaking regions during 2005–2019.

Unsurprisingly, we observed large reductions in VT-type IPD across all age groups after the introduction of both PCV7 and PCV13 in the country, with an earlier and pronounced effect in children <5 years, which has been reported in other studies [17,18]. In contrast, the indirect beneficial effects among age groups, 5–64 years and adults ≥65 years were countered by increasing incidence of non-PCV-type IPD during the late PCV13 era, resulting in an overall IPD incidence that was only 24% and 14% lower, for the respective age groups, compared to the early PCV7 era. Interestingly, the rise in non-PCV-type IPD actually resulted in a rebound of total IPD cases in 2017. This rebound in cases has also been observed in studies from other European countries [18,19]. In England and Wales [18], high influenza activity was reported during the rebound years, which in lieu of an absence of indirect PCV effects, may partly explain the temporary increase in IPD cases. This may also be the case for our Swiss study, though we have yet to analyze the influenza data in detail.

The rising incidence of non-PCV-type IPD after PCV13 introduction, solely among older age groups, especially adults ≥65 years, is concerning. Though these findings are largely consistent with England and Wales [18], studies in the USA have not demonstrated similar trends in their adult population [20]. Lewnard et al. attempted to make sense of these discrepancies and suggested a number of explanations including differences in surveillance sampling, exposure sources and lineage evolution [21]. Of these, we mostly concur that the alarming rise in IPD incidence of some NVTs (8, 22F & 9N) in the aged population, may stem from changes in the usual exposure source (children <5 years), given that these serotypes have been found to be either absent in carriage [22], or carried at a low prevalence (<5%) [23,24,25], suggesting the possibility of other age groups being potential reservoirs or exposure sources for their transmission.

On the national level, our results demonstrated an unclear impact of PCV13 on serotype 3 IPD due to relatively few episodes in children <5 years, and high—albeit fluctuating—incidence among adults ≥65 years. These findings are also consistent with large surveillance studies in France, England and Wales and may hint at a lack of an indirect protection against this serotype in the older age group [18,19]. In Sweden and Portugal, the increased prevalence of serotype 3 in adults ≥65 years was tied to an expansion of a clonal complex, CC180 [26,27], which—as another study showed—had produced a new genetic lineage in the post-PCV13 era with higher antibiotic resistance, recombination rate and antigenic variation than the pre-PCV13 lineages [28]. Antigenic variation may explain the persisting incidence of serotype 3 in adults ≥65 years, but it doesn’t explain its exclusivity to this age group. As for the younger age groups, a recent study recruited children with blood culture-proven sepsis due to *Streptococcus pneumoniae* and it was observed that the incidence of pneumococcal sepsis shortly after introduction of PCV-13 remained substantial [29]. In addition, it was shown that disease caused by serotype 3 represented significant predictors of severity [29]. Further studies are therefore necessary to better understand the immunological response induced by PCV13 against the serotype 3 polysaccharide.

In Switzerland, PCVs are generally well accepted by the population and uptake has reached >80% in more recent years. Based on our data, vaccine uptake is higher in the West (mainly French speaking cantons) compared to the East (German speaking cantons). Recently, a descriptive study based on administrative claims data from a single Swiss health insurer (Helsana) was performed in cohorts of children born between January 2010 and December 2016 [30]. The authors found similar regional differences as outlined in this study and concluded that more emphasis should be placed on timely vaccination [30]. Another recent study in Switzerland demonstrated that teenage girls living in a French-speaking municipality were two times more likely to be vaccinated against HPV compared to those living in the German/Italian-speaking regions [13]. However, after adjusting for other covariates (full model), the authors found that language region was highly correlated with school-based vaccinations and a popular voting result about vaccination laws (vaccine skepticism). We hypothesize that vaccine skepticism may also play a role in the observed regional differences in PCV uptake, however, this warrants further investigation in another study.

Our data shows a correlation of decreased IPD incidence with higher vaccine uptake in the west. However, correlation does not mean causation as factors other than vaccine uptake are also plausible for the observed regional differences in IPD incidences. Among these, clinical practices for investigation and treatment of patients with suspected IPD, as well as case ascertainment and surveillance differences should be considered. Nevertheless, while these variables should be considered if IPD data are compared between nations [18], we believe that given the national guidelines in place, the regional differences for the above explanations will probably have a minimal effect.

Our study has a number of limitations. First, the reported annual incidence of IPD cases among children <5 years, though similar to Sweden [17], is relatively low compared to other countries [18,19,20], which may indicate an under-reporting for mild IPD cases and an underestimation of IPD incidence within this age group in Switzerland [31]. Second, our surveillance dataset had a significant number of IPD cases with unknown ages presenting from the west during 2005–2013 and we could not correct for this confounding. Although this does not affect overall results in both regions, our observation on the indirect herd effects among age group populations during this period in the west should be interpreted with caution. Third, surveillance records did not include information on vaccination history, comorbidities or IPD-related deaths of patients, preventing an in-depth evaluation of serotype-specific association with regions. Lastly, we did not include additional pre-vaccine (2001–2004) data, which may have provided insights on natural fluctuations and temporal trends of circulating pneumococcal strains. However, data from this period has already been examined [32] and we maintain that overall interpretations would remain the same, as we primarily focused on matching periods of vaccine uptake and IPD surveillance.

The strength of our study lies in the use of two highly representative nationwide datasets from the Swiss national vaccination coverage survey and invasive pneumococcal disease surveillance, spanning 15 years of PCV implementation and allowing for a longer post-vaccine follow-up. Also, our IPD surveillance record of serotypes represents a highly uniform methodology via a national reference laboratory with high participation from local laboratories.

## 5. Conclusions

We report an overall decreased IPD incidence across all age groups in Switzerland, with pronounced reductions in children <5 years following the introduction of both PCV7 and PCV13 in the country during 2005–2019. We also observed a predominance of lower IPD incidences in the west associated with higher vaccination coverage compared to the east, though coverage continues to show an overall increasing trend in both regions with decreased IPD incidence. The increasing incidence of non-PCV-type IPD in adults ≥65 years, especially serotypes 8, 22F and 9N, as well as the persistence of serotype 3 in this age group may hint at a need for an exclusive conjugate vaccine for serotypes affecting this group. Continued surveillance therefore remains a priority for early detection of serotype replacement and future vaccine development. At the same time, efforts to further improve vaccination coverage, which at present is >80%, may help further decrease IPD transmission in Switzerland.

## Figures and Tables

**Table 1:** Incidence of invasive pneumococcal disease before and after vaccine introduction in Switzerland, 2005–2019.

	Early PCV7 era Incidence ^a^ (2005–2007)	Late PCV7 era Incidence ^a^ (2008–2010)	Early PCV13 era Incidence ^a^ (2011–2013)	Mid PCV13 era Incidence ^a^ (2014–2016)	Late PCV13 era Incidence ^a^ (2017–2019)	Late PCV7 vs. Early PCV7 IRR ^b^ (95% CI)	Early PCV13 vs. Late PCV7 IRR ^b^ (95% CI)	Mid PCV13 vs. Early PCV13 IRR ^b^ (95% CI)	Late PCV13 vs. Mid PCV13 IRR ^b^ (95% CI)	Late PCV13 vs. Early PCV7 IRR ^b^ (95% CI)
**<5 years**	**n = 219**	**n = 188**	**n = 127**	**n = 91**	**n = 89**					
PCV7	11.1 (9.2–13.2)	3.6 (2.6–4.8)	1.5 (0.9–2.3)	0.6 (0.3–1.2)	0.5 (0.2–1.1)	**0.32 (0.23–0.46)**	**0.42 (0.24–0.72)**	**0.42 (0.19–0.98)**	0.85 (0.31–2.34)	**0.05 (0.02–0.10)**
PCV13nonPCV7	5.4 (4.1–6.9)	9.7 (8.0–11.6)	5.8 (4.5–7.3)	2.5 (1.7–3.6)	2.4 (1.7–3.5)	**1.80 (1.31–2.47)**	**0.60 (0.44–0.80)**	**0.44 (0.29–0.66)**	0.97 (0.60–1.59)	**0.46 (0.30–0.70)**
Non-PCV	3.5 (2.4–4.7)	3.1 (2.2–4.3)	3.2 (2.3–4.4)	4.0 (3.0–5.3)	3.8 (2.8–5.0)	0.91 (0.58–1.43)	1.02 (0.65–1.61)	1.25 (0.82–1.90)	0.95 (0.65–1.41)	1.11 (0.73–1.69)
Total cases	19.9 (17.4–22.7)	16.4 (14.1–18.9)	10.5 (8.7–12.4)	7.2 (5.8–8.8)	6.8 (5.5–8.4)	0.82 (0.68–1.0)	**0.64 (0.51–0.80)**	**0.68 (0.52–0.90)**	0.95 (0.71–1.27)	**0.34 (0.27–0.44)**
**5–64 years**	**n = 1129**	**n = 1308**	**n = 1103**	**n = 879**	**n = 936**					
PCV7	2.6 (2.4–2.9)	2.2 (2.0–2.5)	0.9 (0.8–1.0)	0.4 (0.3–0.5)	0.5 (0.4–0.6)	**0.84 (0.74–0.96)**	**0.40 (0.34–0.48)**	**0.47 (0.36–0.61)**	1.23 (0.91–1.65)	**0.19 (0.16–0.24)**
PCV13nonPCV7	2.2 (2.0–2.5)	2.9 (2.7–3.2)	2.8 (2.5–3.0)	1.5 (1.3–1.6)	1.1 (0.9–1.2)	**1.31 (1.15–1.49)**	0.94 (0.84–1.06)	**0.53 (0.46–0.61)**	**0.73 (0.61–0.88)**	**0.48 (0.41–0.57)**
Non-PCV	1.5 (1.3–1.6)	2.0 (1.8–2.2)	2.2 (2.0–2.5)	2.7 (2.5–2.9)	3.2 (2.9–3.5)	**1.36 (1.16–1.60)**	1.12 (0.97–1.29)	**1.2 (1.06–1.37)**	**1.19 (1.06–1.33)**	**2.18 (1.89–2.52)**
Total cases	6.3 (6.0–6.7)	7.2 (6.8–7.6)	5.9 (5.5–6.2)	4.6 (4.3–4.9)	4.8 (4.5–5.1)	**1.13 (1.04–1.22)**	**0.82 (0.76–0.89)**	**0.78 (0.71–0.85)**	1.05 (0.95–1.15)	**0.75 (0.69–0.82)**
**≥65 years**	**n = 1471**	**n = 1617**	**n = 1518**	**n = 1467**	**n = 1634**					
PCV7	19.7 (18.3–21.2)	14.3 (13.1–15.5)	7.0 (6.3–7.9)	3.8 (3.3–4.5)	2.6 (2.2–3.1)	**0.72 (0.65–0.81)**	**0.50 (0.43–0.57)**	**0.55 (0.45–0.66)**	**0.68 (0.54–0.85)**	**0.13 (0.11–0.16)**
PCV13nonPCV7	11.8 (10.7–13)	14.8 (13.6–16.1)	14.6 (13.5–15.8)	9.6 (8.7–10.5)	8.2 (7.4–9.0)	**1.25 (1.11–1.42)**	0.98 (0.88–1.10)	**0.66 (0.58–0.74)**	**0.85 (0.74–0.98)**	**0.69 (0.60–0.79)**
Non-PCV	8.7 (7.8–9.7)	12.1 (11.0–13.2)	14.6 (13.4–15.8)	19.3 (18.0–20.6)	23.8 (22.4–25.2)	**1.39 (1.21–1.60)**	**1.20 (1.07–1.36)**	**1.32 (1.2–1.47)**	**1.23 (1.13–1.34)**	**2.73 (2.41–3.09)**
Total cases	40.3 (38.2–42.4)	41.2 (39.2–43.2)	36.2 (34.4–38.0)	32.7 (31.1–34.4)	34.5 (32.9–36.2)	1.02 (0.95–1.10)	**0.88 (0.82–0.94)**	**0.90 (0.84–0.97)**	1.06 (0.98–1.13)	**0.86 (0.80–0.92)**
**All ages**	**n = 3031**	**n = 3237**	**n = 2929**	**n = 2612**	**n = 2871**					
PCV7	6.2 (5.9–6.5)	4.5 (4.2–4.7)	2.1 (1.9–2.3)	1.1 (1.0–1.3)	0.9 (0.8–1.1)	**0.72 (0.66–0.78)**	**0.47 (0.42–0.52)**	**0.53 (0.46–0.62)**	**0.83 (0.70–0.99)**	**0.15 (0.13–0.17)**
PCV13nonPCV7	4.3 (4.0–4.6)	5.5 (5.2–5.8)	5.3 (5.0–5.6)	3.2 (2.9–3.4)	2.7 (2.5–2.9)	**1.28 (1.18–1.39)**	0.96 (0.88–1.04)	**0.6 (0.55–0.66)**	**0.85 (0.76–0.94)**	**0.62 (0.56–0.69)**
Non-PCV	2.9 (2.7–3.2)	3.9 (3.7–4.2)	4.8 (4.5–5.1)	6.2 (5.9–6.5)	7.6 (7.3–8.0)	**1.33 (1.21–1.47)**	**1.22 (1.12–1.33)**	**1.3 (1.2–1.4)**	**1.23 (1.15–1.31)**	**2.59 (2.37–2.83)**
Total cases	13.4 (13.0–13.9)	13.9 (13.4–14.3)	12.1 (11.7–12.6)	10.5 (10.1–10.9)	11.2 (10.8–11.6)	1.03 (0.98–1.08)	**0.88 (0.83–0.92)**	**0.86 (0.82–0.91)**	**1.07 (1.02–1.13)**	**0.83 (0.79–0.88)**

^a^ Incidence per 100′000 population (95% CI). ^b^ IRR = Incidence rate ratio. Significant changes in rate ratios in bold. ^c^ Includes cases of unknown age categories (Total unknown = 904).

**Table 2:** Incidence of invasive pneumococcal disease cases (isolates) by region and PCV serotypes, Switzerland 2005–2019.

	IPD Cases- East	IPD Cases- West	Incidence (per 100′000) East	Incidence (per 100′000) West	IRR- West vs. East	95% CI
**2005–2007 (early PCV7 era)**						
PCV7	993	408	6.2	6.1	0.98	0.88–1.11
PCV13nonPCV7	673	292	4.2	4.4	1.04	0.91–1.19
Non-PCV	456	206	2.9	3.1	1.08	0.92–1.28
*Total cases*	2122	906	13.3	13.6	1.02	0.95–1.10
**2008–2010 (late PCV7 era)**						
PCV7	814	225	5.0	3.2	**0.65**	**0.57–0.76**
PCV13nonPCV7	924	355	5.6	5.1	0.91	0.81–1.03
Non-PCV	634	284	3.9	4.1	1.06	0.92–1.22
*Total cases*	2372	864	14.4	12.5	**0.86**	**0.80–0.93**
**2011–2013 (early PCV13 era)**						
PCV7	365	139	2.2	1.9	0.89	0.74–1.09
PCV13nonPCV7	895	376	5.3	5.2	0.99	0.88–1.11
Non-PCV	780	374	4.6	5.2	1.13	1.00–1.27
*Total cases*	2040	889	12.1	12.3	1.02	0.95–1.11
**2014–2016 (mid-PCV13 era)**						
PCV7	226	52	1.3	0.7	**0.54**	**0.40–0.72**
PCV13nonPCV7	585	203	3.3	2.7	**0.81**	**0.69–0.95**
Non-PCV	1110	436	6.4	5.8	0.91	0.82–1.02
*Total cases*	1921	691	11	9.2	**0.84**	**0.77–0.91**
**2017–2019 (late PCV13 era)**						
PCV7	193	44	1.1	0.6	**0.53**	**0.38–0.74**
PCV13nonPCV7	523	159	2.9	2.1	**0.71**	**0.59–0.85**
Non-PCV	1429	514	8.0	6.7	**0.84**	**0.76–0.93**
*Total cases*	2145	717	12	9.3	**0.78**	**0.72–0.85**

West region was defined as primarily French & Italian speaking cantons: Jura (JU), Geneva (GE), Fribourg (FR), Neuchâtel (NE), Valais (VS), Vaud (VD) & Ticino (TI). All other cantons were defined as East. IRR = Incidence rate ratio. Significant changes in IRR are in bold. Serotypes included in PCV7: 4, 6B, 9V, 14, 18C, 19F & 23F. Additional serotypes included in PCV13, but not PCV7: 1, 3, 5, 6A, 7F & 19A. Serotypes not included in either PCV7 or PCV13 were designated as Non-PCV. For 13 isolates/cases, regions were unknown.

## Data Availability

Not applicable.

## Supplementary Materials

The following are available online at https://www.mdpi.com/article/10.3390/microorganisms9051078/s1, Table S1: Proportions of PCV serotypes among IPD, Table S2: Overall incidence of serotypes among IPD, Table S3: Incidence of invasive pneumococcal disease cases in children below 5 years by region and PCV serotypes, Switzerland 2005–2019, Table S4: Incidence of IPD in age group 5–64 years by region and PCV serotypes, Table S5: Incidence of IPD in adults ≥ 65 years by region and PCV serotypes, Table S6: Incidence of serotypes among IPD cases in eastern Switzerland, Table S7: Incidence of serotypes among IPD cases in western Switzerland, 2005–2019. Figure S1: Cantons of Switzerland and their regional classification. Figure S2: Regional estimates of pneumococcal vaccine coverage in children aged 2-years and age-group specific IPD incidence in Switzerland, 2005–2019. (A) regional estimates with up to 1 PCV dose and (B) up to 3 PCV doses, Figure S3: Timeliness of pneumococcal conjugate vaccine uptake in children aged 8 years old in Switzerland and its regions, 2014–2019.
