# Supplementary material for: The Impact of Pneumococcal Conjugate Vaccine (PCV) Coverage Heterogeneities on the Changing Epidemiology of Invasive Pneumococcal Disease in Switzerland, 2005–2019
Source: Microorganisms. 2021 May 18;9(5):1078. doi: 10.3390/microorganisms9051078 (PMC8157260; doi:10.3390/microorganisms9051078)
Supplement: Supplementary file 1 [file microorganisms-09-01078-s001.zip › microorganisms-1189866-supplementary.pdf]

Supplementary Material

Table S1. Proportions of PCV serotypes among invasive pneumococcal disease cases in Switzerland, 2005-2019

**Age category**	**Serotype proportions of IPD cases in different age group populations (%)**															
	**Year**															
	**2005**	**2006**	**2007**	**2008**	**2009**	**2010**	**2011**	**2012**	**2013**	**2014**	**2015**	**2016**	**2017**	**2018**	**2019**	***P*[a]**
< 5 years	45 (4.4)	44 (4.5)	33 (3.2)	21 (1.9)	11 (1)	9 (0.9)	13 (1.3)	2 (0.2)	3 (0.3)	4 (0.5)	0 (0)	4 (0.5)	3 (0.3)	3 (0.3)	1 (0.1)	**< 0.001**
5-64 years	160 (15.7)	145 (14.8)	165 (15.9)	169 (15)	147 (13)	91 (9.3)	69 (6.8)	50 (5.4)	48 (4.9)	32 (3.8)	23 (2.6)	25 (2.9)	36 (3.6)	31 (3.3)	33 (3.6)	**< 0.001**
≥65 years	230 (22.6)	248 (25.4)	243 (23.4)	230 (20.4)	196 (17.4)	134 (13.7)	127 (12.5)	85 (9.2)	83 (8.4)	68 (8)	61 (6.9)	43 (4.9)	41 (4.1)	38 (4)	44 (4.8)	**< 0.001**
Unknown	29 (2.9)	34 (3.5)	26 (2.5)	15 (1.3)	12 (1.1)	8 (0.8)	10 (1)	7 (0.8)	7 (0.7)	9 (1.1)	4 (0.4)	5 (0.6)	5 (0.5)	1 (0.1)	1 (0.1)	**< 0.001**
**Total *PCV7*[b]**	464 (45.7)	471 (48.2)	467 (45)	435 (38.5)	366 (32.5)	242 (24.7)	219 (21.5)	144 (15.6)	141 (14.3)	113 (13.4)	88 (9.9)	77 (8.8)	85 (8.4)	73 (7.7)	79 (8.6)	**< 0.001**
< 5 years	15 (1.5)	19 (1.9)	25 (2.4)	35 (3.1)	44 (3.9)	32 (3.3)	35 (3.4)	21 (2.3)	14 (1.4)	14 (1.7)	10 (1.1)	8 (0.9)	7 (0.7)	18 (1.9)	7 (0.8)	**< 0.001**
5-64 years	135 (13.3)	117 (12)	147 (14.2)	155 (13.7)	203 (18)	179 (18.2)	189 (18.5)	158 (17.1)	171 (17.4)	99 (11.7)	103 (11.6)	80 (9.1)	74 (7.3)	79 (8.4)	58 (6.3)	**< 0.001**
≥65 years	139 (13.7)	134 (13.7)	159 (15.3)	188 (16.7)	195 (17.3)	199 (20.3)	217 (21.3)	207 (22.4)	188 (19.1)	144 (17)	138 (15.5)	147 (16.8)	146 (14.4)	130 (13.8)	110 (12)	0.2
Unknown	27 (2.7)	23 (2.4)	27 (2.6)	18 (1.6)	20 (1.8)	13 (1.3)	19 (1.9)	36 (3.9)	16 (1.6)	12 (1.4)	21 (2.4)	12 (1.4)	24 (2.4)	15 (1.6)	16 (1.7)	0.2
***Total PCV13nonPCV7*[c]**	316 (31.1)	293 (30)	358 (34.5)	396 (35.1)	462 (41)	423 (43.1)	460 (45.1)	422 (45.7)	389 (39.5)	269 (31.8)	272 (30.6)	247 (28.2)	251 (24.8)	242 (25.6)	191 (20.9)	**< 0.001**
< 5 years	10 (1)	14 (1.4)	14 (1.4)	7 (0.6)	12 (1.1)	17 (1.7)	11 (1.1)	15 (1.6)	13 (1.3)	15 (1.8)	20 (2.2)	16 (1.8)	17 (1.7)	17 (1.8)	16 (1.7)	**0.005**
5-64 years	89 (8.8)	84 (8.6)	87 (8.4)	120 (10.6)	127 (11.3)	117 (11.9)	121 (11.9)	128 (13.9)	169 (17.2)	144 (17)	177 (19.9)	196 (22.3)	215 (21.2)	203 (21.5)	207 (22.6)	**< 0.001**
≥65 years	119 (11.7)	95 (9.7)	104 (10)	155 (13.7)	149 (13.2)	171 (17.4)	187 (18.3)	179 (19.4)	245 (24.9)	270 (31.9)	292 (32.8)	304 (34.7)	391 (38.6)	359 (38)	375 (41)	**< 0.001**
Unknown	18 (1.8)	21 (2.1)	7 (0.7)	16 (1.4)	11 (1)	11 (1.1)	22 (2.2)	36 (3.9)	28 (2.8)	35 (4.1)	40 (4.5)	37 (4.2)	53 (5.2)	50 (5.3)	47 (5.1)	**< 0.001**
***Total Non-PCV*[d]**	236 (23.2)	214 (21.9)	212 (20.4)	298 (26.4)	299 (26.4)	316 (32.2)	341 (33.4)	358 (38.7)	455 (46.2)	464 (54.8)	529 (59.5)	553 (63.1)	676 (66.8)	629 (66.6)	645 (70.5)	**< 0.001**
**All cases**	**1016 (100)**	**978 (100)**	**1037 (100)**	**1129 (100)**	**1127 (100)**	**981 (100)**	**1020 (100)**	**924 (100)**	**985 (100)**	**846 (100)**	**889 (100)**	**877 (100)**	**1012 (100)**	**944 (100)**	**915 (100)**	

a *P*-values for chi-squared test of trend. $P < 0.05$ indicated in bold.
b Serotypes included in PCV7: 4, 6B, 9V, 14, 18C, 19F & 23F
c Additional serotypes included in PCV13, but not PCV7: 1, 3, 5, 6A, 7F & 19A
d Serotypes not included in either PCV7 or PCV13

Table S2. Overall incidence of serotypes among IPD cases in Switzerland, 2005-2019

**Serotype/ serogroup**[a]	**Incidence of serotypes/serogroups among IPD cases in Switzerland (Cases per 100'000 population)**														
	**Year**														
	**2005**	**2006**	**2007**	**2008**	**2009**	**2010**	**2011**	**2012**	**2013**	**2014**	**2015**	**2016**	**2017**	**2018**	**2019**
**4**	77 (1)	81 (1.1)	67 (0.9)	79 (1)	79 (1)	51 (0.6)	48 (0.6)	32 (0.4)	27 (0.3)	21 (0.3)	18 (0.2)	19 (0.2)	21 (0.2)	13 (0.2)	8 (0.1)
**6B**	48 (0.6)	38 (0.5)	32 (0.4)	26 (0.3)	27 (0.3)	16 (0.2)	22 (0.3)	11 (0.1)	7 (0.1)	7 (0.1)	4 (0)	5 (0.1)	9 (0.1)	4 (0)	4 (0)
**9V**	73 (1)	57 (0.8)	77 (1)	68 (0.9)	58 (0.7)	29 (0.4)	19 (0.2)	13 (0.2)	19 (0.2)	6 (0.1)	7 (0.1)	7 (0.1)	6 (0.1)	8 (0.1)	12 (0.1)
**14**	136 (1.8)	151 (2)	142 (1.9)	111 (1.4)	88 (1.1)	66 (0.8)	56 (0.7)	30 (0.4)	39 (0.5)	37 (0.4)	29 (0.3)	19 (0.2)	17 (0.2)	19 (0.2)	22 (0.3)
**18C**	25 (0.3)	35 (0.5)	39 (0.5)	30 (0.4)	19 (0.2)	23 (0.3)	17 (0.2)	10 (0.1)	9 (0.1)	12 (0.1)	4 (0)	4 (0)	1 (0)	6 (0.1)	8 (0.1)
**19F**	44 (0.6)	49 (0.7)	50 (0.7)	48 (0.6)	34 (0.4)	29 (0.4)	29 (0.4)	17 (0.2)	27 (0.3)	21 (0.3)	16 (0.2)	20 (0.2)	27 (0.3)	18 (0.2)	23 (0.3)
**23F**	61 (0.8)	60 (0.8)	60 (0.8)	73 (0.9)	61 (0.8)	28 (0.4)	28 (0.4)	31 (0.4)	13 (0.2)	9 (0.1)	10 (0.1)	3 (0)	4 (0)	5 (0.1)	2 (0)
**1**	60 (0.8)	40 (0.5)	37 (0.5)	50 (0.6)	49 (0.6)	57 (0.7)	49 (0.6)	41 (0.5)	26 (0.3)	24 (0.3)	6 (0.1)	5 (0.1)	3 (0)	0 (0)	1 (0)
**3**	121 (1.6)	108 (1.4)	135 (1.8)	151 (2)	162 (2.1)	126 (1.6)	168 (2.1)	137 (1.7)	163 (2)	131 (1.6)	151 (1.8)	163 (1.9)	158 (1.9)	187 (2.2)	149 (1.7)
**6A**	20 (0.3)	40 (0.5)	36 (0.5)	37 (0.5)	45 (0.6)	30 (0.4)	20 (0.3)	24 (0.3)	18 (0.2)	18 (0.2)	11 (0.1)	7 (0.1)	5 (0.1)	4 (0)	4 (0)
**7F**	83 (1.1)	80 (1.1)	98 (1.3)	99 (1.3)	115 (1.5)	109 (1.4)	106 (1.3)	99 (1.2)	96 (1.2)	43 (0.5)	37 (0.4)	26 (0.3)	22 (0.3)	9 (0.1)	6 (0.1)
**19A**	30 (0.4)	24 (0.3)	43 (0.6)	49 (0.6)	79 (1)	99 (1.3)	116 (1.5)	119 (1.5)	82 (1)	50 (0.6)	66 (0.8)	46 (0.5)	63 (0.7)	42 (0.5)	31 (0.4)
**10A**	0 (0)	0 (0)	1 (0)	1 (0)	1 (0)	9 (0.1)	16 (0.2)	15 (0.2)	19 (0.2)	20 (0.2)	22 (0.3)	23 (0.3)	24 (0.3)	25 (0.3)	30 (0.3)
**11A**	0 (0)	0 (0)	0 (0)	3 (0)	2 (0)	13 (0.2)	23 (0.3)	15 (0.2)	18 (0.2)	25 (0.3)	22 (0.3)	20 (0.2)	27 (0.3)	24 (0.3)	19 (0.2)
**12F**	0 (0)	0 (0)	3 (0)	11 (0.1)	9 (0.1)	5 (0.1)	8 (0.1)	16 (0.2)	17 (0.2)	19 (0.2)	21 (0.3)	25 (0.3)	29 (0.3)	37 (0.4)	48 (0.6)
**15B/C**	0 (0)	1 (0)	11 (0.1)	10 (0.1)	15 (0.2)	14 (0.2)	11 (0.1)	17 (0.2)	13 (0.2)	20 (0.2)	19 (0.2)	23 (0.3)	24 (0.3)	25 (0.3)	1 (0)
**15A**	0 (0)	0 (0)	0 (0)	9 (0.1)	5 (0.1)	5 (0.1)	10 (0.1)	8 (0.1)	10 (0.1)	20 (0.2)	20 (0.2)	24 (0.3)	31 (0.4)	27 (0.3)	29 (0.3)
**20**	4 (0.1)	3 (0)	8 (0.1)	14 (0.2)	10 (0.1)	9 (0.1)	10 (0.1)	6 (0.1)	8 (0.1)	6 (0.1)	21 (0.3)	10 (0.1)	17 (0.2)	13 (0.2)	12 (0.1)
**22F**	0 (0)	0 (0)	30 (0.4)	33 (0.4)	45 (0.6)	61 (0.8)	62 (0.8)	54 (0.7)	85 (1)	92 (1.1)	66 (0.8)	85 (1)	91 (1.1)	86 (1)	103 (1.2)
**23A**	0 (0)	1 (0)	4 (0.1)	11 (0.1)	12 (0.2)	8 (0.1)	11 (0.1)	11 (0.1)	17 (0.2)	24 (0.3)	19 (0.2)	16 (0.2)	27 (0.3)	20 (0.2)	29 (0.3)
**23B**	0 (0)	0 (0)	4 (0.1)	6 (0.1)	3 (0)	8 (0.1)	8 (0.1)	13 (0.2)	23 (0.3)	22 (0.3)	33 (0.4)	20 (0.2)	39 (0.5)	33 (0.4)	23 (0.3)
**24**	2 (0)	1 (0)	10 (0.1)	5 (0.1)	9 (0.1)	3 (0)	7 (0.1)	15 (0.2)	22 (0.3)	25 (0.3)	42 (0.5)	31 (0.4)	15 (0.2)	13 (0.2)	0 (0)
**35F**	0 (0)	0 (0)	6 (0.1)	7 (0.1)	8 (0.1)	17 (0.2)	4 (0.1)	15 (0.2)	18 (0.2)	14 (0.2)	20 (0.2)	16 (0.2)	15 (0.2)	16 (0.2)	20 (0.2)
**6C**	0 (0)	0 (0)	4 (0.1)	8 (0.1)	12 (0.2)	21 (0.3)	23 (0.3)	16 (0.2)	40 (0.5)	10 (0.1)	26 (0.3)	20 (0.2)	22 (0.3)	15 (0.2)	13 (0.2)
**8**	57 (0.8)	57 (0.8)	32 (0.4)	50 (0.6)	51 (0.7)	45 (0.6)	60 (0.8)	53 (0.7)	66 (0.8)	60 (0.7)	89 (1.1)	107 (1.3)	150 (1.8)	160 (1.9)	142 (1.7)
**9N**	0 (0)	0 (0)	27 (0.4)	41 (0.5)	26 (0.3)	32 (0.4)	32 (0.4)	33 (0.4)	31 (0.4)	31 (0.4)	32 (0.4)	55 (0.7)	64 (0.8)	50 (0.6)	63 (0.7)
**others**[b]	175 (2.3)	152 (2)	81 (1.1)	99 (1.3)	103 (1.3)	68 (0.9)	57 (0.7)	73 (0.9)	72 (0.9)	79 (1)	78 (0.9)	78 (0.9)	101 (1.2)	85 (1)	113 (1.3)
Total	**1016 (13.6)**	**978 (13)**	**1037 (13.7)**	**1129 (14.7)**	**1127 (14.5)**	**981 (12.5)**	**1020 (12.8)**	**924 (11.5)**	**985 (12.1)**	**846 (10.3)**	**889 (10.7)**	**877 (10.4)**	**1012 (11.9)**	**944 (11)**	**915 (10.6)**

a Serotypes/serogroups with proportions ≥1% are highlighted as such: PCV7 in peach, additional serotypes included in PCV13, but not PCV7 are in blue and non-PCV serotypes are in gray.

b Serotypes/serogroups with proportions <1% were classified as others irrespective of PCV type.

Table S3. Incidence of invasive pneumococcal disease cases in children below 5 years by region and PCV serotypes, Switzerland 2005-2019

Children <5 years	IPD cases- East	IPD cases- West	Incidence (per 100'000) East	Incidence (per 100'000) West	IRR- West vs. East	95% CI
**2005-2007 (early PCV7 era)**						
**PCV7**	85	37	11.2	10.9	0.97	0.66-1.43
**PCV13nonPCV7**	47	12	6.2	3.5	0.57	0.30-1.07
**Non-PCV**	33	5	4.3	1.5	**0.34**	**0.13-0.87**
**Total cases**	165	54	21.7	15.8	**0.73**	**0.54-0.99**
**2008-2010 (late PCV7 era)**						
**PCV7**	34	7	4.3	2	0.46	0.20-1.04
**PCV13nonPCV7**	72	39	9.1	11	1.21	0.82-1.79
**Non-PCV**	22	14	2.8	3.9	1.42	0.73-2.78
**Total cases**	128	60	16.1	16.9	1.05	0.77-1.42
**2011-2013 (early PCV13 era)**						
**PCV7**	11	7	1.3	1.9	1.45	0.56-3.74
**PCV13nonPCV7**	55	15	6.5	4	0.62	0.35-1.10
**Non-PCV**	27	12	3.2	3.2	1.01	0.51-2.00
**Total cases**	93	34	11	9.2	0.83	0.56-1.23
**2014-2016 (mid PCV13 era)**						
**PCV7**	6	2	0.7	0.5	0.77	0.16-3.82
**PCV13nonPCV7**	28	4	3.2	1	**0.33**	**0.12-0.94**
**Non-PCV**	39	12	4.4	3.1	0.71	0.37-1.36
**Total cases**	73	18	8.2	4.7	**0.57**	**0.34-0.96**
**2017-2019 (late PCV13 era)**						
**PCV7**	5	2	0.5	0.5	0.94	0.18-4.86
**PCV13nonPCV7**	30	2	3.3	0.5	**0.16**	**0.04-0.66**
**Non-PCV**	36	14	3.9	3.6	0.92	0.50-1.70
**Total cases**	71	18	7.7	4.6	0.60	0.36-1.00

West region was defined as primarily French & Italian speaking cantons: Jura (JU), Geneva (GE), Fribourg (FR), Neuchâtel (NE), Valais (VS), Vaud (VD) & Ticino (TI). All other cantons were defined as East. IRR= Incidence rate ratio. Significant changes in IRR are in bold. Serotypes included in PCV7: 4, 6B, 9V, 14, 18C, 19F & 23F. Additional serotypes included in PCV13, but not PCV7: 1, 3, 5, 6A, 7F & 19A. Serotypes not included in either PCV7 or PCV13 were designated as Non-PCV. For 13 isolates/cases, regions were unknown.

Table S4. Incidence of invasive pneumococcal disease cases in age group 5-64 years by region and PCV serotypes, Switzerland 2005-2019

**5-64 years**	**IPD cases- East**	**IPD cases- West**	**Incidence (per 100'000) East**	**Incidence (per 100'000) West**	**IRR- West vs. East**	**95% CI**
**2005-2007 (early PCV7 era)**						
**PCV7**	323	147	2.6	2.8	1.09	0.90-1.33
**PCV13nonPCV7**	281	117	2.2	2.2	1.00	0.81-1.24
**Non-PCV**	189	71	1.5	1.4	0.90	0.69-1.18
**Total cases**	793	335	6.3	6.4	1.01	0.89-1.15
**2008-2010 (late PCV7 era)**						
**PCV7**	312	94	2.4	1.7	**0.71**	**0.57-0.90**
**PCV13nonPCV7**	401	135	3.1	2.5	**0.80**	**0.66-0.97**
**Non-PCV**	251	115	2.0	2.1	1.08	0.87-1.35
**Total cases**	964	344	7.5	6.3	**0.84**	**0.75-0.95**
**2011-2013 (early PCV13 era)**						
**PCV7**	121	46	0.9	0.8	0.89	0.63-1.25
**PCV13nonPCV7**	375	143	2.9	2.6	0.89	0.74-1.08
**Non-PCV**	297	121	2.3	2.2	0.95	0.77-1.18
**Total cases**	793	310	6	5.5	0.92	0.80-1.04
**2014-2016 (mid PCV13 era)**						
**PCV7**	69	11	0.5	0.2	**0.37**	**0.20-0.70**
**PCV13nonPCV7**	206	76	1.5	1.3	0.85	0.66-1.11
**Non-PCV**	385	132	2.9	2.3	**0.79**	**0.65-0.97**
**Total cases**	660	219	4.9	3.8	**0.77**	**0.66-0.89**
**2017-2019 (late PCV13 era)**						
**PCV7**	88	12	0.6	0.2	**0.31**	**0.17-0.58**
**PCV13nonPCV7**	160	51	1.2	0.9	0.74	0.54-1.01
**Non-PCV**	467	155	3.4	2.6	**0.77**	**0.64-0.92**
**Total cases**	715	218	5.2	3.7	**0.70**	**0.61-0.82**

West region was defined as primarily French & Italian speaking cantons: Jura (JU), Geneva (GE), Fribourg (FR), Neuchâtel (NE), Valais (VS), Vaud (VD) & Ticino (TI). All other cantons were defined as East. IRR= Incidence rate ratio. Significant changes in IRR are in bold. Serotypes included in PCV7: 4, 6B, 9V, 14, 18C, 19F & 23F. Additional serotypes included in PCV13, but not PCV7: 1, 3, 5, 6A, 7F & 19A. Serotypes not included in either PCV7 or PCV13 were designated as Non-PCV. For 13 isolates/cases, regions were unknown.

Table S5. Incidence of invasive pneumococcal disease cases in adults ≥ 65 years by region and PCV serotypes, Switzerland 2005-2019

**≥65 years**	**IPD cases-East**	**IPD cases-West**	**Incidence (per 100'000) East**	**Incidence (per 100'000) West**	**IRR- West vs. East**	**95% CI**
**2005-2007 (early PCV7 era)**						
**PCV7**	549	171	21.2	16.1	**0.76**	**0.64-0.91**
**PCV13nonPCV7**	316	115	12.2	10.9	0.89	0.72-1.10
**Non-PCV**	218	100	8.4	9.4	1.12	0.89-1.42
**Total cases**	1083	386	41.7	36.4	**0.87**	**0.78-0.98**
**2008-2010 (late PCV7 era)**						
**PCV7**	449	108	16.1	9.4	**0.58**	**0.47-0.72**
**PCV13nonPCV7**	439	143	15.8	12.5	**0.79**	**0.66-0.96**
**Non-PCV**	348	129	12.5	11.3	0.90	0.74-1.10
**Total cases**	1236	380	44.4	33.2	**0.75**	**0.67-0.84**
**2011-2013 (early PCV13 era)**						
**PCV7**	218	77	7.3	6.3	0.85	0.66-1.11
**PCV13nonPCV7**	443	169	14.9	13.7	0.92	0.77-1.10
**Non-PCV**	425	186	14.3	15.1	1.06	0.89-1.26
**Total cases**	1086	432	36.6	35.1	0.96	0.86-1.07
**2014-2016 (mid PCV13 era)**						
**PCV7**	139	33	4.4	2.5	**0.57**	**0.39-0.83**
**PCV13nonPCV7**	331	98	10.5	7.4	**0.71**	**0.57-0.89**
**Non-PCV**	632	234	20	17.8	0.89	0.77-1.03
**Total cases**	1102	365	34.8	27.7	**0.80**	**0.71-0.90**
**2017-2019 (late PCV13 era)**						
**PCV7**	96	27	2.9	2.0	0.68	0.45-1.05
**PCV13nonPCV7**	308	77	9.2	5.6	**0.61**	**0.47-0.78**
**Non-PCV**	852	270	25.4	19.6	**0.77**	**0.67-0.88**
**Total cases**	1256	374	37.5	27.1	**0.72**	**0.65-0.81**

West region was defined as primarily French & Italian speaking cantons: Jura (JU), Geneva (GE), Fribourg (FR), Neuchâtel (NE), Valais (VS), Vaud (VD) & Ticino (TI). All other cantons were defined as East. IRR= Incidence rate ratio. Significant changes in IRR are in bold. Serotypes included in PCV7: 4, 6B, 9V, 14, 18C, 19F & 23F. Additional serotypes included in PCV13, but not PCV7: 1, 3, 5, 6A, 7F & 19A. Serotypes not included in either PCV7 or PCV13 were designated as Non-PCV. For 13 isolates/cases, regions were unknown.

Table S6. Incidence of serotypes among IPD cases in eastern Switzerland, 2005-2019

**Serotype/ serogroup[a]**	**Incidence of serotypes/serogroups among IPD cases in the East[b] (Cases per 100'000 population)**														
	**Year**														
	**2005**	**2006**	**2007**	**2008**	**2009**	**2010**	**2011**	**2012**	**2013**	**2014**	**2015**	**2016**	**2017**	**2018**	**2019**
**4**	57 (1.1)	49 (0.9)	47 (0.9)	59 (1.1)	63 (1.2)	36 (0.7)	31 (0.6)	21 (0.4)	13 (0.2)	19 (0.3)	17 (0.3)	19 (0.3)	20 (0.3)	12 (0.2)	8 (0.1)
**6B**	30 (0.6)	29 (0.5)	26 (0.5)	19 (0.4)	24 (0.4)	15 (0.3)	16 (0.3)	7 (0.1)	7 (0.1)	7 (0.1)	3 (0.1)	1 (0)	8 (0.1)	2 (0)	4 (0.1)
**9V**	50 (0.9)	44 (0.8)	52 (1)	51 (0.9)	45 (0.8)	23 (0.4)	14 (0.3)	7 (0.1)	13 (0.2)	4 (0.1)	3 (0.1)	4 (0.1)	5 (0.1)	6 (0.1)	11 (0.2)
**14**	93 (1.8)	121 (2.3)	98 (1.8)	81 (1.5)	71 (1.3)	52 (0.9)	43 (0.8)	23 (0.4)	27 (0.5)	28 (0.5)	26 (0.4)	16 (0.3)	13 (0.2)	14 (0.2)	20 (0.3)
**18C**	19 (0.4)	24 (0.5)	30 (0.6)	22 (0.4)	15 (0.3)	21 (0.4)	14 (0.3)	9 (0.2)	7 (0.1)	11 (0.2)	4 (0.1)	3 (0.1)	1 (0)	6 (0.1)	7 (0.1)
**19F**	34 (0.6)	31 (0.6)	31 (0.6)	37 (0.7)	27 (0.5)	22 (0.4)	22 (0.4)	13 (0.2)	21 (0.4)	17 (0.3)	15 (0.3)	12 (0.2)	17 (0.3)	17 (0.3)	15 (0.2)
**23F**	33 (0.6)	44 (0.8)	51 (1)	58 (1.1)	53 (1)	23 (0.4)	24 (0.4)	21 (0.4)	12 (0.2)	7 (0.1)	7 (0.1)	3 (0.1)	3 (0.1)	2 (0)	2 (0)
**1**	31 (0.6)	28 (0.5)	25 (0.5)	35 (0.6)	32 (0.6)	42 (0.8)	27 (0.5)	29 (0.5)	19 (0.3)	21 (0.4)	4 (0.1)	4 (0.1)	3 (0.1)	0 (0)	1 (0)
**3**	84 (1.6)	75 (1.4)	91 (1.7)	111 (2)	133 (2.4)	94 (1.7)	115 (2.1)	90 (1.6)	125 (2.2)	96 (1.7)	103 (1.8)	123 (2.1)	120 (2)	140 (2.3)	124 (2.1)
**6A**	15 (0.3)	28 (0.5)	30 (0.6)	28 (0.6)	36 (0.7)	21 (0.4)	17 (0.3)	22 (0.4)	16 (0.3)	16 (0.3)	9 (0.2)	7 (0.1)	4 (0.1)	1 (0)	4 (0.1)
**7F**	65 (1.2)	56 (1.1)	73 (1.4)	71 (1.3)	88 (1.6)	75 (1.4)	80 (1.4)	68 (1.2)	74 (1.3)	36 (0.6)	32 (0.5)	21 (0.4)	19 (0.3)	7 (0.1)	5 (0.1)
**19A**	20 (0.4)	16 (0.3)	28 (0.5)	32 (0.6)	58 (1.1)	56 (1)	75 (1.3)	76 (1.3)	55 (1)	35 (0.6)	47 (0.8)	27 (0.5)	42 (0.7)	31 (0.5)	22 (0.4)
**10A**	0 (0)	0 (0)	1 (0)	1 (0)	0 (0)	6 (0.1)	8 (0.1)	9 (0.2)	14 (0.2)	10 (0.2)	16 (0.3)	17 (0.3)	17 (0.3)	15 (0.3)	18 (0.3)
**11A**	0 (0)	0 (0)	0 (0)	2 (0)	2 (0)	11 (0.2)	16 (0.3)	7 (0.1)	14 (0.2)	13 (0.2)	15 (0.3)	15 (0.3)	21 (0.4)	20 (0.3)	15 (0.2)
**12F**	0 (0)	0 (0)	3 (0.1)	11 (0.2)	4 (0.1)	5 (0.1)	7 (0.1)	10 (0.2)	11 (0.2)	17 (0.3)	16 (0.3)	16 (0.3)	18 (0.3)	32 (0.5)	39 (0.6)
**15B/C**	0 (0)	0 (0)	8 (0.1)	10 (0.2)	10 (0.2)	8 (0.1)	2 (0)	9 (0.2)	7 (0.1)	15 (0.3)	16 (0.3)	19 (0.3)	17 (0.3)	21 (0.4)	1 (0)
**15A**	0 (0)	0 (0)	0 (0)	7 (0.1)	1 (0)	2 (0)	4 (0.1)	2 (0)	8 (0.1)	15 (0.3)	16 (0.3)	13 (0.2)	24 (0.4)	17 (0.3)	14 (0.2)
**22F**	0 (0)	0 (0)	22 (0.4)	19 (0.4)	36 (0.7)	49 (0.9)	45 (0.8)	38 (0.7)	67 (1.2)	65 (1.1)	47 (0.8)	62 (1.1)	66 (1.1)	69 (1.2)	86 (1.4)
**23A**	0 (0)	0 (0)	2 (0)	8 (0.1)	7 (0.1)	5 (0.1)	6 (0.1)	9 (0.2)	11 (0.2)	18 (0.3)	15 (0.3)	8 (0.1)	19 (0.3)	16 (0.3)	20 (0.3)
**23B**	0 (0)	0 (0)	3 (0.1)	4 (0.1)	0 (0)	3 (0.1)	5 (0.1)	7 (0.1)	18 (0.3)	16 (0.3)	29 (0.5)	18 (0.3)	31 (0.5)	23 (0.4)	17 (0.3)
**24**	2 (0)	1 (0)	9 (0.2)	2 (0)	6 (0.1)	0 (0)	7 (0.1)	9 (0.2)	14 (0.2)	18 (0.3)	34 (0.6)	29 (0.5)	10 (0.2)	11 (0.2)	0 (0)
**35F**	0 (0)	0 (0)	4 (0.1)	6 (0.1)	6 (0.1)	12 (0.2)	4 (0.1)	10 (0.2)	11 (0.2)	8 (0.1)	11 (0.2)	12 (0.2)	9 (0.2)	13 (0.2)	13 (0.2)
**6C**	0 (0)	0 (0)	3 (0.1)	4 (0.1)	8 (0.1)	14 (0.3)	14 (0.3)	12 (0.2)	25 (0.4)	9 (0.2)	20 (0.3)	12 (0.2)	19 (0.3)	12 (0.2)	8 (0.1)
**8**	35 (0.7)	36 (0.7)	20 (0.4)	31 (0.6)	34 (0.6)	30 (0.5)	39 (0.7)	27 (0.5)	47 (0.8)	41 (0.7)	63 (1.1)	69 (1.2)	104 (1.8)	109 (1.8)	101 (1.7)
**9N**	0 (0)	0 (0)	22 (0.4)	36 (0.7)	19 (0.3)	19 (0.3)	25 (0.4)	24 (0.4)	22 (0.4)	23 (0.4)	23 (0.4)	39 (0.7)	50 (0.8)	37 (0.6)	53 (0.9)
**others[c]**	126 (2.4)	105 (2)	62 (1.2)	72 (1.3)	79 (1.4)	54 (1)	49 (0.9)	59 (1)	55 (1)	66 (1.1)	73 (1.3)	57 (1)	79 (1.3)	78 (1.3)	87 (1.4)
Total*	**694 (13.2)**	**687 (13)**	**741 (13.8)**	**817 (15.1)**	**857 (15.7)**	**698 (12.6)**	**709 (12.7)**	**618 (11)**	**713 (12.5)**	**631 (10.9)**	**664 (11.4)**	**626 (10.6)**	**739 (12.5)**	**711 (11.9)**	**695 (11.5)**

a Serotypes/serogroups with proportions ≥1% are highlighted as such: PCV7 in peach, additional serotypes included in PCV13, but not PCV7 are in blue and non-PCV serotypes are in gray.
b East region defined as primarily German speaking cantons in Switzerland.
c Serotypes/serogroups with proportions <1% were classified as others irrespective of PCV type.
* Excludes 13 isolates/cases with unknown regional classification

Table S7. Incidence of serotypes among IPD cases in western Switzerland, 2005-2019

Serotype/ serogroup[a]	Incidence of serotypes/serogroups among IPD cases in the West[b] (Cases per 100'000 population)														
	**Year**														
	**2005**	**2006**	**2007**	**2008**	**2009**	**2010**	**2011**	**2012**	**2013**	**2014**	**2015**	**2016**	**2017**	**2018**	**2019**
**4**	20 (0.9)	32 (1.4)	20 (0.9)	20 (0.9)	16 (0.7)	15 (0.6)	17 (0.7)	11 (0.5)	14 (0.6)	2 (0.1)	1 (0)	0 (0)	1 (0)	1 (0)	0 (0)
**6B**	18 (0.8)	9 (0.4)	6 (0.3)	7 (0.3)	3 (0.1)	1 (0)	6 (0.3)	4 (0.2)	0 (0)	0 (0)	1 (0)	4 (0.2)	1 (0)	2 (0.1)	0 (0)
**9V**	23 (1.1)	13 (0.6)	25 (1.1)	17 (0.7)	13 (0.6)	6 (0.3)	5 (0.2)	6 (0.3)	6 (0.2)	2 (0.1)	4 (0.2)	3 (0.1)	1 (0)	2 (0.1)	1 (0)
**14**	43 (2)	30 (1.4)	44 (2)	30 (1.3)	17 (0.7)	14 (0.6)	13 (0.5)	7 (0.3)	12 (0.5)	9 (0.4)	3 (0.1)	3 (0.1)	4 (0.2)	5 (0.2)	2 (0.1)
**18C**	6 (0.3)	11 (0.5)	9 (0.4)	8 (0.4)	4 (0.2)	2 (0.1)	3 (0.1)	1 (0)	2 (0.1)	1 (0)	0 (0)	1 (0)	0 (0)	0 (0)	1 (0)
**19F**	10 (0.5)	18 (0.8)	19 (0.8)	11 (0.5)	7 (0.3)	7 (0.3)	7 (0.3)	4 (0.2)	6 (0.2)	4 (0.2)	1 (0)	8 (0.3)	10 (0.4)	1 (0)	8 (0.3)
**23F**	28 (1.3)	15 (0.7)	9 (0.4)	15 (0.7)	8 (0.3)	5 (0.2)	4 (0.2)	10 (0.4)	1 (0)	2 (0.1)	3 (0.1)	0 (0)	1 (0)	3 (0.1)	0 (0)
**1**	29 (1.3)	12 (0.5)	12 (0.5)	15 (0.7)	17 (0.7)	15 (0.6)	22 (0.9)	12 (0.5)	7 (0.3)	3 (0.1)	2 (0.1)	1 (0)	0 (0)	0 (0)	0 (0)
**3**	37 (1.7)	32 (1.4)	44 (2)	40 (1.8)	29 (1.3)	32 (1.4)	53 (2.2)	47 (2)	38 (1.6)	35 (1.4)	48 (1.9)	40 (1.6)	38 (1.5)	47 (1.8)	24 (0.9)
**6A**	5 (0.2)	12 (0.5)	6 (0.3)	9 (0.4)	9 (0.4)	9 (0.4)	3 (0.1)	2 (0.1)	2 (0.1)	2 (0.1)	2 (0.1)	0 (0)	1 (0)	3 (0.1)	0 (0)
**7F**	18 (0.8)	23 (1)	25 (1.1)	28 (1.2)	27 (1.2)	34 (1.5)	26 (1.1)	31 (1.3)	22 (0.9)	7 (0.3)	5 (0.2)	5 (0.2)	3 (0.1)	2 (0.1)	1 (0)
**19A**	10 (0.5)	8 (0.4)	15 (0.7)	17 (0.7)	21 (0.9)	43 (1.8)	41 (1.7)	43 (1.8)	27 (1.1)	15 (0.6)	19 (0.8)	19 (0.8)	21 (0.8)	11 (0.4)	8 (0.3)
**10A**	0 (0)	0 (0)	0 (0)	0 (0)	1 (0)	3 (0.1)	8 (0.3)	6 (0.3)	5 (0.2)	10 (0.4)	6 (0.2)	6 (0.2)	7 (0.3)	10 (0.4)	12 (0.5)
**11**	5 (0.2)	8 (0.4)	6 (0.3)	13 (0.6)	6 (0.3)	2 (0.1)	1 (0)	0 (0)	0 (0)	0 (0)	0 (0)	0 (0)	0 (0)	0 (0)	0 (0)
**11A**	0 (0)	0 (0)	0 (0)	1 (0)	0 (0)	2 (0.1)	7 (0.3)	8 (0.3)	4 (0.2)	12 (0.5)	7 (0.3)	5 (0.2)	6 (0.2)	4 (0.2)	3 (0.1)
**12F**	0 (0)	0 (0)	0 (0)	0 (0)	5 (0.2)	0 (0)	1 (0)	6 (0.3)	6 (0.2)	2 (0.1)	5 (0.2)	9 (0.4)	11 (0.4)	5 (0.2)	9 (0.3)
**15B/C**	0 (0)	1 (0)	3 (0.1)	0 (0)	5 (0.2)	6 (0.3)	9 (0.4)	8 (0.3)	6 (0.2)	5 (0.2)	3 (0.1)	4 (0.2)	7 (0.3)	4 (0.2)	0 (0)
**15A**	0 (0)	0 (0)	0 (0)	2 (0.1)	4 (0.2)	3 (0.1)	6 (0.3)	6 (0.3)	2 (0.1)	5 (0.2)	4 (0.2)	11 (0.4)	7 (0.3)	10 (0.4)	15 (0.6)
**20**	0 (0)	2 (0.1)	2 (0.1)	6 (0.3)	2 (0.1)	0 (0)	2 (0.1)	1 (0)	2 (0.1)	2 (0.1)	6 (0.2)	6 (0.2)	8 (0.3)	4 (0.2)	7 (0.3)
**22F**	0 (0)	0 (0)	8 (0.4)	14 (0.6)	9 (0.4)	12 (0.5)	17 (0.7)	16 (0.7)	18 (0.7)	27 (1.1)	19 (0.8)	23 (0.9)	25 (1)	17 (0.7)	16 (0.6)
**23A**	0 (0)	1 (0)	2 (0.1)	3 (0.1)	5 (0.2)	3 (0.1)	5 (0.2)	2 (0.1)	6 (0.2)	6 (0.2)	4 (0.2)	8 (0.3)	8 (0.3)	4 (0.2)	9 (0.3)
**23B**	0 (0)	0 (0)	1 (0)	2 (0.1)	3 (0.1)	5 (0.2)	3 (0.1)	6 (0.3)	5 (0.2)	6 (0.2)	4 (0.2)	2 (0.1)	8 (0.3)	10 (0.4)	6 (0.2)
**24**	0 (0)	0 (0)	1 (0)	3 (0.1)	3 (0.1)	3 (0.1)	0 (0)	6 (0.3)	8 (0.3)	7 (0.3)	8 (0.3)	2 (0.1)	5 (0.2)	2 (0.1)	0 (0)
**35F**	0 (0)	0 (0)	2 (0.1)	1 (0)	2 (0.1)	5 (0.2)	0 (0)	5 (0.2)	7 (0.3)	6 (0.2)	9 (0.4)	4 (0.2)	6 (0.2)	3 (0.1)	7 (0.3)
**6C**	0 (0)	0 (0)	1 (0)	4 (0.2)	4 (0.2)	7 (0.3)	9 (0.4)	4 (0.2)	15 (0.6)	1 (0)	6 (0.2)	8 (0.3)	3 (0.1)	3 (0.1)	5 (0.2)
**8**	22 (1)	21 (0.9)	12 (0.5)	19 (0.8)	17 (0.7)	15 (0.6)	21 (0.9)	26 (1.1)	19 (0.8)	19 (0.8)	26 (1)	38 (1.5)	46 (1.8)	50 (1.9)	41 (1.6)
**9N**	0 (0)	0 (0)	5 (0.2)	5 (0.2)	7 (0.3)	13 (0.6)	7 (0.3)	9 (0.4)	9 (0.4)	8 (0.3)	9 (0.4)	16 (0.6)	14 (0.5)	13 (0.5)	10 (0.4)
**others[c]**	48 (2.2)	40 (1.8)	19 (0.8)	21 (0.9)	26 (1.1)	21 (0.9)	15 (0.6)	19 (0.8)	23 (0.9)	17 (0.7)	20 (0.8)	25 (1)	31 (1.2)	15 (0.6)	28 (1.1)
Total*	**322 (14.7)**	**288 (13)**	**296 (13.2)**	**311 (13.6)**	**270 (11.7)**	**283 (12.1)**	**311 (13.1)**	**306 (12.8)**	**272 (11.2)**	**215 (8.7)**	**225 (9)**	**251 (9.9)**	**273 (10.7)**	**231 (9)**	**213 (8.3)**

a Serotypes/serogroups with proportions ≥1% are highlighted as such: PCV7 in peach, additional serotypes included in PCV13, but not PCV7 are in blue and non-PCV serotypes are in gray.
b West region defined as primarily French and Italian speaking cantons in Switzerland.
c Serotypes/serogroups with proportions <1% were classified as others irrespective of PCV type.
***** Excludes 13 isolates/cases with unknown regional classification.

Supplementary Figures

Figure S1. Cantons of Switzerland and their regional classification

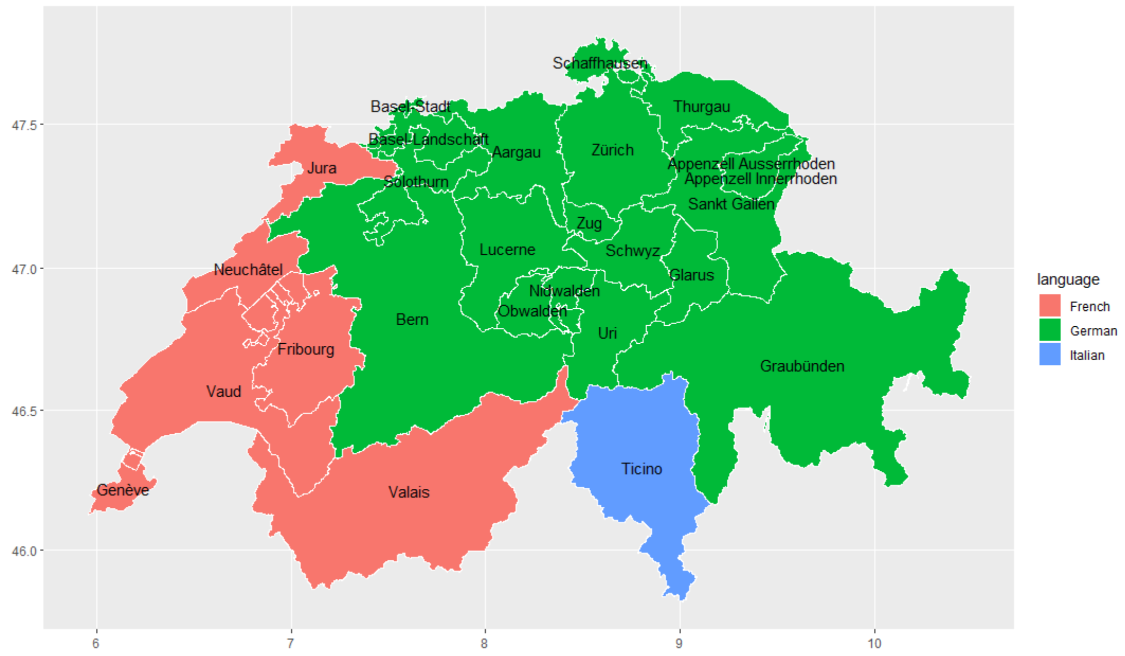

Canton(s)	Majority Language	Regional classification
**Ticino (TI)**	**Italian**	**West**
**Fribourg (FR), Geneva (GE), Jura (JU), Neuchâtel (NE), Valais (VS), Vaud (VD)**	**French**	**West**
**Aargau (AG), Appenzell Ausserrhoden (AR), Appenzell Innerrhoden (AI), Basel-Landschaft (BL), Basel-Stadt (BS), Bern (BE), Glarus (GL), Graubünden (GR), Lucerne (LU), Nidwalden (NW), Obwalden (OW), Schaffhausen (SH), Schwyz (SZ), Solothurn (SO), St. Gallen (SG), Thurgau (TG), Uri (UR), Zug (ZG), Zürich (ZH).**	**German**	**East**

Figure S2. Regional estimates of pneumococcal vaccine coverage in children aged 2-years and age-group specific IPD incidence in Switzerland, 2005-2019. (A) regional estimates with up to 1 PCV dose and (B) up to 3 PCV doses.

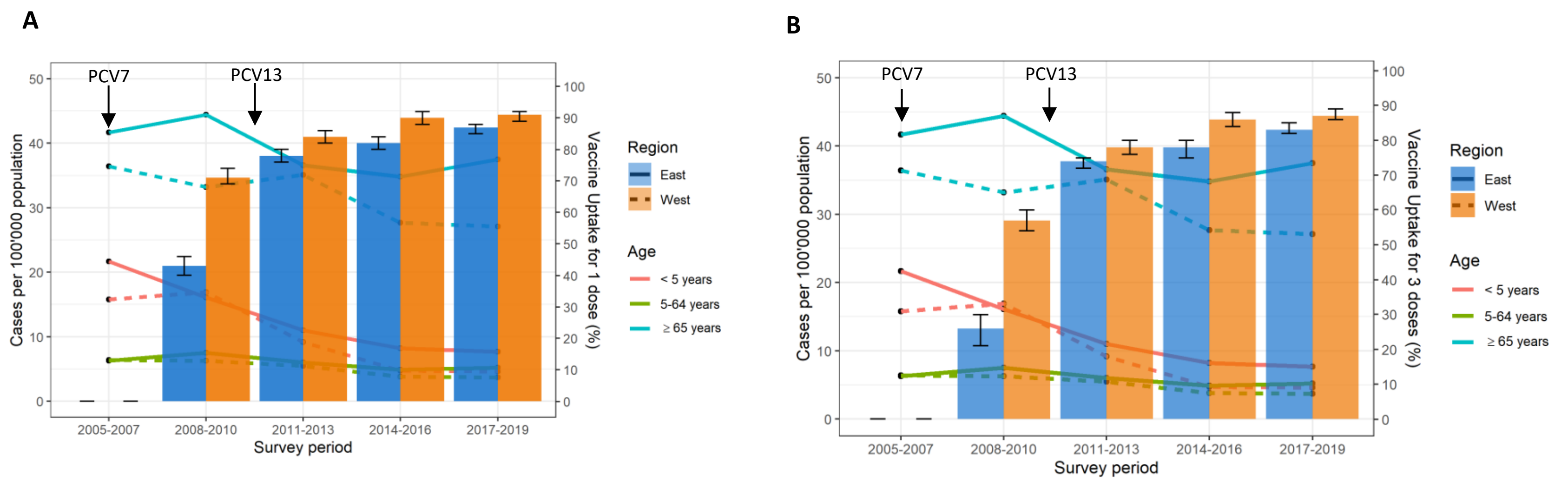

Figure S3. Timeliness of pneumococcal conjugate vaccine uptake in children aged 8 years old in Switzerland and its regions, 2014-2019.

(A) Timeliness of vaccine uptake in children aged 8 years vaccinated with 1-3 doses of PCV7 or PCV13, (B) regional estimates of pneumococcal vaccination coverage among children 8 years with up to 1 PCV dose, (C) up to 2 PCV doses and (D) up to 3 PCV doses.

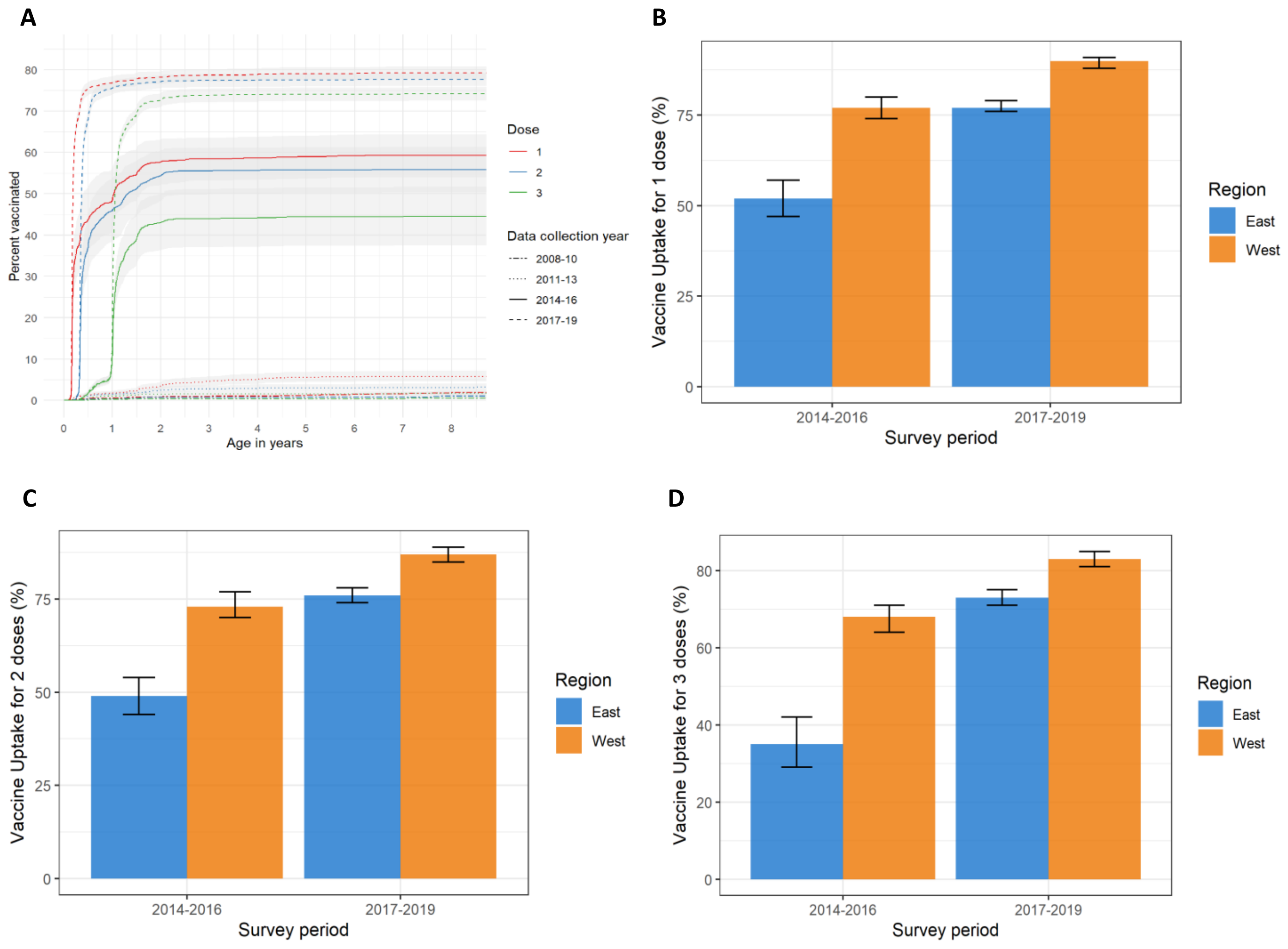
A
Percent vaccinated
Age in years
Dose
1
2
3
Data collection year
2008-10
2011-13
2014-16
2017-19
B
Vaccine Uptake for 1 dose (%)
Survey period
2014-2016
2017-2019
Region
East
West
C
Vaccine Uptake for 2 doses (%)
Survey period
2014-2016
2017-2019
Region
East
West
D
Vaccine Uptake for 3 doses (%)
Survey period
2014-2016
2017-2019
Region
East
West
